# AI-driven integration and optimization of medicinal plant multi-omics metabolic networks

**DOI:** 10.3389/fpls.2026.1756809

**Published:** 2026-03-31

**Authors:** Jun Chen, Jinyu Cai, Hong To Quyen Duong, Somnuk Bunsupa, Rongchun Han, Xiaohui Tong

**Affiliations:** 1 School of Pharmacy, Anhui University of Chinese Medicine, Hefei, China; 2 Scientific Research - Healthcare Activity Direction Department, Traditional Medicine Hospital of Ho Chi Minh City, Ho Chi Minh City, Vietnam; 3 Faculty of Pharmacy, Mahidol University, Bangkok, Thailand; 4 School of Life Sciences, Anhui University of Chinese Medicine, Hefei, China

**Keywords:** artificial intelligence, deep learning, medicinal plant, multi-omics, secondary metabolite

## Abstract

Natural products from medicinal plants are vital sources for medicines, but understanding their complex production pathways within the plant is challenging. This review explores how artificial intelligence (AI), defined here as a suite of computational techniques including Machine Learning (ML), Deep Learning (DL), Natural Language Processing (NLP), and network analysis—is transforming this field of research. We describe how AI technologies, particularly machine and deep learning, are used to integrate large, heterogeneous biological datasets, extract features and identify key components in the biosynthesis of valuable compounds, and model how these metabolic networks behave over time. The review demonstrates that AI technologies effectively integrate large biological datasets to model dynamic metabolic behaviors. Furthermore, AI facilitates the optimization of the entire production chain, from cultivation conditions to extraction parameters. Ultimately, these technologies are shifting the research paradigm from conventional methods to precise, data-driven approaches, accelerating the sustainable bioproduction of plant-based natural products.

## 1 Introduction

Natural products, also known as specialized metabolites, have historically been vital sources for pharmaceuticals and agrochemicals. These compounds are biosynthesized through complex pathways in medicinal plants. Understanding these pathways is crucial to harness nature’s synthetic capacity and enables sustainable bioproduction of valuable compounds. However, research on plant secondary metabolism faces major challenges (Rai et al., 2017), including multi-omics data fragmentation, reliance on manual pathway inference, and difficulties in capturing dynamic, nonlinear regulatory mechanisms within metabolic networks.

Traditional methods for analyzing biosynthetic pathways primarily rely on correlation-based statistical tools such as Pearson correlation analysis, Principal Component Analysis (PCA), and Weighted Gene Co-expression Network Analysis (WGCNA). These methods have limitations in inferring causality and combinatorial interactions. For instance, although integrated multi-omics identified key enzymatic steps in hypericin biosynthesis in *Hypericum perforatum*, the absence of dynamic modeling hindered metabolic flux prediction (Karalija et al., 2025). Similarly, Alami et al. (2022) noted persistent reliance on correlation analysis despite multi-omics advances.

Before AI’s widespread application, constraint-based models showed promise. Murali et al. (2023) constructed the first genome-scale model for *Nothapodytes nimmoniana* identifying targets that increased camptothecin yield fivefold upon validation, though such models struggle with dynamic processes like Methyl Jasmonate (MeJA) induction.

Artificial intelligence now offers transformative potential. To provide clarity on the scope and application of AI in this domain, we define it in the context of this review as an umbrella concept encompassing a suite of computational methods that enable machines to simulate intelligent behavior, particularly in learning, reasoning, and decision-making, when processing complex biological data. ML, DL, and NLP models effectively integrate multi-omics data and predict enzyme function, particularly excelling in handling time-series data to capture dynamic responses like MeJA-induced metabolic changes (Shi et al., 2021). Deep learning methods have been increasingly applied to integrate multi-omics data. For instance, autoencoders can learn compressed, shared latent representations from heterogeneous datasets. In contrast, Graph Convolutional Networks (GCNs) operate directly on graph-structured data, where nodes represent biological entities (e.g., genes, proteins, metabolites) and edges represent their functional relationships (e.g., co-expression, protein-protein interactions). Through iterative message-passing between nodes, GCNs generate context-aware embeddings that capture both the attributes of individual entities and the topological structure of the underlying biological network. This makes them particularly suitable for modeling complex metabolic pathways (Yang et al., 2020). Meanwhile, ensemble tree-based models, such as the AutoGluon-Tabular framework used by Bai et al. (2024), have demonstrated superior performance in static multi-class prediction of biosynthetic gene families by integrating genomic and proteomic features. Metabolomics Data Integration and Translation (MetDIT) method (Sha et al., 2024) converts metabolomic data to images using Convolutional Neural Networks (CNNs), outperforming traditional ML, while Kang et al. (2021) demonstrated deep learning’s advantage in handling high-dimensional multi-omics data.

We further delineate the core AI technologies and map their respective roles onto a generalized workflow of “Data → Knowledge → Design → Optimization” to elucidate their transformative impact:

In terms of Data Integration and Preprocessing, ML and DL methods form the foundation for processing heterogeneous, high-dimensional multi-omics data. For example, DL-based integration roadmaps provide a framework for standardizing and fusing data from different platforms (Kang et al., 2021), while specific studies have effectively achieved correlation analysis between transcriptomic and metabolomic data through standardized workflows (Zhang et al., 2024).

In terms of Feature Extraction and Pattern Recognition, Supervised Machine Learning excels at screening key biomarkers from high-dimensional data, such as identifying crucial features influencing phenolic synthesis from metabolomics data (García-Pérez et al., 2021) or predicting component content from hyperspectral data (Dai et al., 2024). Deep Learning models, particularly CNNs and Deep Neural Networks (DNNs), are more adept at capturing complex nonlinear relationships, applied in tasks such as species classification (Jiang et al., 2025) or spatiotemporal expression pattern analysis.

In terms of Network Construction and Dynamic Modeling, Graph Neural Networks (GNNs) play a central role in this phase, naturally modeling biological entities (genes, proteins, metabolites) and their relationships as graph structures to construct and analyze gene-enzyme-metabolite regulatory networks (Yang et al., 2020). Artificial Neural Networks (ANNs) have been successfully used to simulate dynamic changes in metabolic processes, such as modeling the signal response dynamics in taxol biosynthesis (Gong et al., 2007). However, the application of Convolutional Neural Networks (CNNs) to omics data is not straightforward, as raw 1D feature vectors (e.g., from metabolomics) lack the spatial or sequential locality that CNNs are designed to exploit. To address this limitation, methods such as the MetDIT framework (Sha et al., 2024) first transform 1D metabolomic profiles into 2D image-like representations (e.g., via Gramian Angular Fields), thereby creating pseudo-spatial correlations that CNNs can then leverage for tasks such as sample classification or biomarker discovery. This conversion step is critical: it aligns the data structure with the inductive bias of the CNN, enabling its powerful feature extraction capabilities.

In terms of Pathway Inference and Optimization, Knowledge Graphs (KGs) and NLP techniques support enzyme function prediction and pathway backtracking by integrating domain knowledge and mining literature (Fu et al., 2023). Reinforcement Learning (RL) and optimization strategies combined with Genetic Algorithms (GAs) are employed to explore optimal solutions within vast engineering design spaces, such as optimizing extraction processes (Rakshit and Srivastav, 2021) or predicting ideal cultivation conditions (Hu et al., 2025).

By systematically applying this AI technology stack, researchers can overcome two key limitations: the inability of traditional methods to infer causality and combinatorial interactions (Alami et al., 2022), and the difficulty of constraint-based models in capturing dynamic processes (Murali et al., 2023). This approach creates a closed loop from data to actionable design solutions.

This review explores the crucial role of AI in advancing biosynthetic pathway research and its potential to accelerate the discovery of natural products. It outlines the contributions of ML, DL, NLP, network analysis, and data mining techniques in efficiently parsing vast biological datasets. Finally, the review summarizes the challenges faced by the field and future directions, emphasizing the significant advancements and promising prospects of AI in biosynthetic pathway research.

## 2 Review methodology

**Figure 1:**
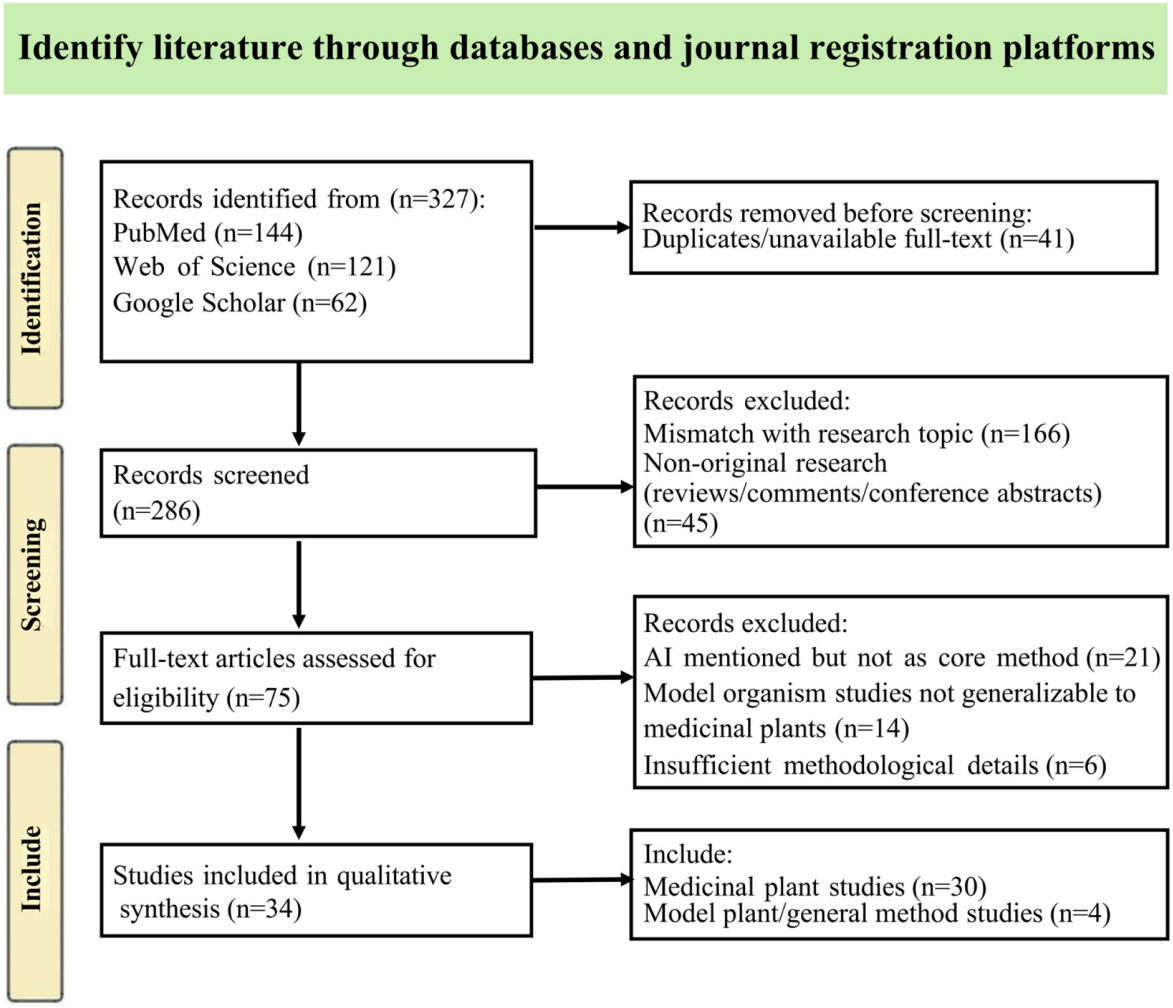
Literature screening procedure.

To ensure the comprehensiveness, objectivity, and reproducibility of this review, we employed a systematic literature search and analysis strategy, adhering to the methodological framework of systematic reviews. The complete literature screening process is presented in **Figure 1**.

### 2.1 Literature search strategy

We systematically searched the following electronic databases: PubMed, Web of Science Core Collection, and Google Scholar. The search period was restricted from January 2000 to December 2025, encompassing early explorations of artificial intelligence in medicinal plant metabolism research (e.g., the pioneering work by Gong et al. (2007) on ANNs for simulating taxol biosynthesis dynamics) through the most recent advances (e.g., the conference paper by Zhao et al. (2025) on multi-omics studies of ginseng).

Search terms were grouped into three core concepts as follows:

1. Artificial intelligence-related terms: “artificial intelligence”, “machine learning”, “deep learning”, “neural networks”, “artificial neural network (ANN)”, “convolutional neural network (CNN)”, “graph neural network (GNN)”, “graph convolutional network (GCN)”, “graph attention network (GAT)”, “deep neural network (DNN)”, “multilayer perceptron (MLP)”, “support vector machine (SVM)”, “random forest”, “genetic algorithm”, “reinforcement learning”, “natural language processing”, “knowledge graph”, “physics-informed neural network (PINN)”
2. Medicinal plant and metabolism-related terms: “medicinal plant”, “herbal medicine”, “traditional Chinese medicine”, “plant secondary metabolism”, “specialized metabolism”, “biosynthetic pathway”, “secondary metabolite”, “natural product”, “*Taxus chinensis*”, “*Panax ginseng*”, “*Salvia miltiorrhiza*”, “*Glycyrrhiza uralensis*”, “*Hypericum perforatum*”, “*Angelica biserrata*”, “*Pithecellobium dulce*”, “*Bryophyllum*”, “chamomile”, “opium poppy”, “pomegranate”
3. Multi-omics and network-related terms: “multi-omics”, “genomics”, “transcriptomics”, “proteomics”, “metabolomics”, “microRNAome”, “metabolic network”, “gene-enzyme-metabolite network”, “data integration”, “pathway analysis”, “metabolic engineering”, “feature extraction”, “pattern recognition”, “time-series analysis”, “dynamic modeling”, “metabolic flux”, “genome-scale model”

In addition, we manually screened the reference lists of included articles and citations from relevant high-quality reviews to identify any potentially eligible studies missed during the initial database search.

### 2.2 Literature inclusion and exclusion criteria

Studies were included or excluded based on the following pre-established criteria:

Inclusion criteria: 1) Original research articles or authoritative review papers published in peer-reviewed journals. 2) Studies explicitly applying AI technologies to elucidate biosynthetic pathways or optimize metabolic networks in medicinal plants. 3) Studies incorporating at least one type of omics data or involving metabolic network analysis. 4) Studies providing clear descriptions of AI models, their application contexts, and/or quantitative validation results (e.g., performance metrics such as R², AUC, accuracy). 5) For studies involving key regulator screening, inclusion of the number of candidates identified and/or experimental validation data. 6) Full-text availability and publication in English or Chinese.

Exclusion criteria:

1. Conference abstracts, commentaries, letters, or patent literature. (Note: Zhao et al. (2025), a conference paper, was included due to its substantial contributions to ginseng multi-omics research.)
2. Studies focused exclusively on model organisms (e.g., *Arabidopsis thaliana*) or microbial systems, where the methodologies and findings cannot be reasonably extrapolated to medicinal plant research.
3. Articles that mention AI techniques only superficially without employing them as core analytical methods.
4. Duplicate publications.
5. Studies lacking sufficient methodological detail for critical evaluation.
6. Purely descriptive studies that do not report performance evaluation of the AI models employed.

Special Note: While this review primarily focuses on medicinal plants, we acknowledge the foundational role of model organisms (e.g., *Arabidopsis thaliana*) in advancing metabolic network research and AI methodology development. Given the potential translational value of these methodological innovations, we selectively included a limited number of studies conducted in model plants that offer significant methodological insights applicable to medicinal plant research. Such inclusions were justified only when the proposed AI method or analytical framework demonstrated sufficient generality for direct transfer to medicinal plant studies [e.g., the graph attention network for metabolic pathway prediction by Yang et al. (2020); the AutoGluon-Tabular multi-omics feature selection approach by Bai et al. (2024)]. All such studies are explicitly identified in the main text as providing methodological insights, ensuring clear differentiation from studies yielding medicinal plant-specific conclusions.

### 2.3 Data extraction and analysis framework

From included studies, we extracted: plant species, target metabolites, AI models, application scenarios, findings, performance metrics, and validation strategies. Data were organized by AI roles within the “Data Integration → Knowledge Discovery → Design Optimization → Application Validation” pipeline, consistent with the Introduction’s framework.

### 2.4 Literature screening process

Initial search yielded 327 records. After duplicate removal, 286 remained. Title/abstract screening excluded 211 irrelevant records, leaving 75 for full-text assessment. Full-text evaluation against criteria resulted in 34 included studies. The screening process is illustrated in **Figure 1**.

## 3 Core approaches of AI technology in processing multi-omics data of medicinal plants

The integration of multi-omics technologies in medicinal plant research has generated complex, high-dimensional datasets that challenge conventional analytical methods. Artificial intelligence, particularly ML and DL, provides powerful solutions for processing and integrating these heterogeneous data to reconstruct biosynthetic networks of secondary metabolites.

### 3.1 Preprocessing and standardization of multi-omics data

High-quality preprocessing is essential for reliable analysis. A primary hurdle in multi-omics analysis is the heterogeneity of data derived from disparate platforms (transcriptomics, proteomics, metabolomics). These datasets often exhibit significant batch effects and scale variations. Therefore, rigorous quality control and standardization are prerequisite steps to ensure the reliability of subsequent AI modeling. For example, in a study on *Salvia miltiorrhiza* under drought stress (Zhang et al., 2024), transcriptomic and metabolomic data from aerial and underground tissues were standardized to eliminate scale effects, enabling effective cross-omics correlation analysis. As emphasized in deep learning-based integration roadmaps (Kang et al., 2021), preprocessing is an indispensable step for building reliable predictive models.

### 3.2 Feature extraction and pattern recognition

After standardization, extracting key biological features from high-dimensional data becomes critical. ML excels in this regard: in studies on *Bryophyllum* (García-Pérez et al., 2021), untargeted metabolomics combined with ML identified crucial metabolite features influencing phenolic synthesis, leading to high-accuracy predictive models. Similarly, in *S. miltiorrhiza* research (Dai et al., 2024), ML strategies selected characteristic wavelengths from hyperspectral data, enabling accurate prediction of tanshinone content and geographical origin classification.

DL models perform particularly well with complex, nonlinear multi-omics data. In *Panax ginseng* studies (Zhao et al., 2025), convolutional neural networks analyzed spatiotemporal gene expression dynamics to identify key regulatory modules for secondary metabolite synthesis. Models like Deep Graph of Waters (DeepGOW) predicted interactions between licorice components and severe acute respiratory syndrome coronavirus 2 (SARS-CoV-2) targets, validating known compounds and discovering new active molecules (Fu et al., 2023). Deep neural networks applied to metabolomic data also achieved 100% accuracy in classifying *P. ginseng* species (Jiang et al., 2025), outperforming traditional ML methods. This result was rigorously validated via five-fold cross-validation and comprehensive benchmarking against RF, SVM, XGBoost, and MLP.

While the studies above demonstrate the broad applicability of ML and DL in feature extraction, a critical comparison reveals important distinctions in model suitability that are often overlooked in proof-of-concept reports. For instance, in the *Bryophyllum* study (García-Pérez et al., 2021), traditional ML algorithms such as Random Forest achieved high predictive accuracy with relatively small sample sizes (n < 100), highlighting their strength in scenarios where interpretability, low computational cost, and resistance to overfitting are prioritized. In contrast, the CNN applied to Panax ginseng (Zhao et al., 2025) excelled in capturing spatiotemporal expression patterns. However, it required substantially larger training datasets (n > 500) and careful architecture tuning to avoid overfitting, a common limitation when transferring DL methods to medicinal plant systems where multi-omics data remain scarce and heterogeneous. Similarly, the DeepGOW model (Fu et al., 2023) demonstrated the power of graph-based reasoning for compound-target prediction, yet its reliance on pre-existing knowledge graphs (e.g., KEGG, STITCH) limits its utility for under-studied medicinal species with limited prior annotations. These comparisons underscore a central methodological insight: no single AI method is universally optimal for feature extraction in medicinal plant research. Instead, model selection must be guided by data availability, the complexity of the biological question, and the trade-off between predictive performance and interpretability. To move the field forward, future studies should systematically benchmark multiple models on the same dataset, and ideally make their code and data publicly available. This would provide clearer, evidence-based guidelines for method selection and help shift the field from descriptive case studies toward generalizable analytical frameworks.

### 3.3 Multi-omics data correlation analysis and network construction

A key goal of AI is to integrate multi-omics layers into gene–enzyme–metabolite regulatory networks to elucidate cross-omics regulatory logic. This requires models capable of capturing complex, heterogeneous relationships. Multimodal deep learning frameworks and GNNs have emerged as pivotal tools for this task. Multimodal frameworks, such as those using cross-modal attention mechanisms, can learn aligned representations from different omics modalities. GNNs naturally model biological systems as graphs (Yang et al., 2020), where nodes represent entities (genes, proteins, metabolites) and edges represent interactions or correlations, making them exceptionally suitable for integrating multi-omics data and inferring network topology. Both supervised and unsupervised methods contribute significantly: Orthogonal Projections to Latent Structures-Discriminant Analysis (OPLS-DA) distinguished metabolic profiles under different conditions and identified key classification metabolites (García-Pérez et al., 2021), while artificial neural networks optimized *chamomile* extraction by modeling nonlinear relationships between parameters and phenolic content (Cvetanović Kljakić et al., 2023). The ESP model used gradient boosting trees to predict enzyme–substrate interactions (Lim et al., 2023), facilitating the construction of association networks. Graph deep learning frameworks, such as graph attention networks (Yang et al., 2020), treated molecular structures as graphs to classify metabolic pathways accurately.

Additionally, AI supports predicting drug–herb interactions (Spanakis et al., 2025), forecasting Absorption, Distribution, Metabolism, Excretion, and Toxicology (ADME-Tox) properties of metabolites (Ramya et al., 2022), and mining literature associations via natural language processing. CNNs and RNNs (Sha et al., 2024) have also been used to analyze transcriptome time-series and extract discriminative metabolomic features.

In summary, artificial intelligence technologies have demonstrated their transformative capacity in deciphering the complexity of multi-omics data from medicinal plants. Specifically, through machine learning and deep learning methods, AI systematically addresses core challenges such as data heterogeneity, high dimensionality, and nonlinearity. The workflow begins with strategic data preprocessing and fusion, employing early, intermediate, or late fusion strategies tailored to specific biological questions (Kang et al., 2021). The process then advances to feature extraction and pattern recognition. For instance, supervised machine learning excels at high-dimensional biomarker screening, such as selecting characteristic wavelengths from hyperspectral data to predict tanshinone content (Dai et al., 2024). Meanwhile, deep learning captures complex nonlinear relationships, as demonstrated using convolutional neural networks to analyze spatiotemporal gene expression dynamics in *Panax ginseng* (Zhao et al., 2025). The culmination of this integration process is network construction and dynamic modeling, where multimodal deep learning frameworks and graph neural networks play pivotal roles. They can align different omics layers (e.g., genomics, transcriptomics, metabolomics) and reconstruct gene-enzyme-metabolite regulatory networks (Zhao et al., 2025), thereby elucidating cross-omics regulatory logic.

This AI-driven pipeline transforms fragmented data into actionable insights, laying the foundation for metabolic engineering. Crucially, constructing detailed metabolic maps is not an end, but a means to a higher goal: the systematic navigation and optimization of these networks. Integrated multi-omics data thus provides the essential raw material, while clear objectives, such as enhancing metabolite yield, give the models purpose and direction. These two components form a synergistic, iterative research cycle, propelling discovery from descriptive understanding toward predictive design and practical application.

Building on this integrated foundation, AI-driven modeling and optimization now aim to rationally design and enhance these biosynthetic pathways.

To provide a systematic overview of how different AI models have been applied in medicinal plant metabolism research, **Table 1** summarizes representative studies by model type, highlighting their advantages and limitations.

**Table 1:** Quantitative comparison of AI models with validation and cost-benefit metrics.

AI model type	Reference	Plant/compound studied	Application and function	Performance validation results	Validation requirement cost-benefit
ANN	Gong et al., 2007	*Taxus chinensis*	A five-layer feedforward ANN was constructed to simulate ROS dynamics and predict taxol yield using time and elicitor concentration as inputs.	R = 0.9997 vs. experimental ROS measurements; taxol yield prediction error <5%	Requirements: 18 time points (vs. 54 for ODE models); elicitor gradient data Cost-Benefit: 60% reduction in consumables; 3x faster model development
GNN	Zhao et al., 2025	*Panax ginseng*	Integrated multi-omics data to construct gene–enzyme–metabolite networks and simulate metabolic flux variations	Network completion 92.3% vs. WGCNA 68%: flux simulation 85% agreement with experiments; identified 27 key regulators	Requirements: Genome annotation ($5,000-10,000); paired transcriptome-metabolome data Cost-Benefit: 80% reduction in manual curation; saves $20,000-30,000 in validation costs
CNN	Sha et al., 2024; Dai et al., 2024	*Salvia miltiorrhiza*; *Panax ginseng*	MetDIT framework transformed metabolomics data into image-like representations for analysis; hyperspectral imaging predicted compound content	Classification accuracy 100% (3,872 features) vs. SVM 92% and RF 85%; tanshinone prediction R^2^ = 0.94	Requirements: MS data ($200/sample) or hyperspectral imaging; minimum 30 training samples Cost-Benefit: No feature engineering (saves 2–3 weeks); 10x faster analysis; 70% lower cost per sample
SVM	Durand et al., 2024; Bai et al., 2024	*Papaver somniferum*; *Arabidopsis thaliana*	Used for enzyme function prediction, gene classification, and feature selection	AUC-ROC=0.85-0.89 vs. logistic regression 0.72-0.78; enzyme prediction accuracy 87%; 5-fold CV stable	Requirements: 50–100 training samples (50% less than DL); annotated functional gene data Cost-Benefit: 90% candidate reduction; runs on standard PC; ideal for preliminary screening
Deep Learning Ensemble Model	Bai et al., 2024	*Arabidopsis thaliana*	Automated multi-omics feature selection to identify biosynthetic genes	AUC-ROC > 0.89; top 50 candidates achieve 30-40% experimental validation rate; identified 12 new genes, 5 confirmed	Requirements: Minimum 500 training genes; pair multi-omics data Cost-Benefit: 10,000x enrichment vs. random screening; validation cost $5,000 per target (vs.$50,000 traditional); 90% cost reduction
Graph Attention Network (GAT)	Yang et al., 2020	general metabolic pathway prediction	Treated molecular structures as graphs for pathway classification	Classification accuracy 92.3% vs. fingerprint methods 78-85%; KEGG test set 91.7%; interpretable substructure identification	Requirements: Molecular structure annotations (free from PubChem); KEGG pathway labels Cost-Benefit: No experimental data needed for novel compounds; 70% faster database search; 50x higher throughput
Deep Learning + Bayesian Optimization	Hu et al., 2025	*Angelica biserrata*	Predicted coumarin accumulation under varying environmental conditions (R² = 0.977)	R^2^ = 0.977 (test set) vs. MLR 0.68-0.75; field validation 92% accuracy; identified 3 key environmental factors	Requirements: 2 years field data; minimum 50 sampling sites Cost-Benefit: 50-60% reduction in field trials; 23% yield increase; $300–500 additional income per acre
ANN + GA	Rakshit and Srivastav, 2021	Pomegranate peel	Optimized pulsed ultrasound-assisted extraction via multi-objective optimization	23% yield increase vs. RSM; R^2^ = 0.97 vs. RSM 0.89; Pareto front identified 15 feasible solutions	Requirements: 18 experiments (vs. 30 for RSM); 3 process parameters Cost-Benefit: 40% fewer experiments; 35% consumable savings (~$2,000); 50% shorter optimization (4wksizatio
Multilayer Perceptron (MLP)	Saffariha et al., 2021	*Hypericum perforatum*	Predicted hypericin content based on 13 ecological factors (test R² = 0.90)	Test R^2^ = 0.90 vs. habitat models 0.60-0.70; field validation error <15%; identified phenology and soil carbon as key	Requirements: 13 ecological factors; minimum 50 training sites Cost-Benefit: 70% reduction in field survey costs (~$8,000 savings); rapid site screening; < $10 per prediction

## 4 AI-Driven modeling and optimization of medicinal plant metabolic networks

AI is integrating into medicinal plant research with unprecedented depth and breadth, providing a powerful methodology for parsing biosynthetic pathways, dynamically simulating, and systematically optimizing core secondary metabolites. By integrating multi-omics data, AI-driven strategies enable leaps from metabolic network structural modeling and key regulator identification to full-chain optimization, significantly accelerating the process of medicinal plant metabolic engineering.

### 4.1 Structural modeling and dynamic simulation of metabolic networks

Constructing precise metabolic network models is fundamental for understanding and regulating metabolic flux. GNNs and CNN show unique advantages here. Based on graph representation learning and knowledge graph reasoning methods, it is possible to integrate complex associations among genes, proteins, and metabolites to automatically infer and complete missing links in metabolic pathways. This approach continues the early computational biology tradition of formalizing and automatically reasoning about metabolic networks, exemplified by the “predecessor list” representation and rule-based reaction direction inference system proposed by Karp and Paley (1994) which successfully reconstructed accurate metabolic pathway diagrams from minimal data. The pioneering work by Gong et al. (2007) demonstrated the potential of artificial neural networks for dynamic simulation in medicinal plant systems. They constructed a five-layer feedforward network using time and elicitor concentration as inputs to successfully simulate the reactive oxygen species (ROS) burst dynamics in *Taxus chinensis* cells induced by a bio-elicitor. The model exhibited excellent agreement with experimental data (R = 0.9997 between simulated and measured ROS levels), enabling the identification of three distinct phases of ROS accumulation. The study also captured the relationship between ROS levels and final taxol yield, serving as an early proof-of-concept for using neural networks to explore complex signal regulation mechanisms in plant secondary metabolism and laying a methodological foundation for subsequent research. This model revealed that cells employ an integral feedback control mechanism for precise signal response regulation, providing key insights for using ANNs to analyze complex biological signal transduction networks.

At a broader network construction level, GNNs are specifically used to integrate multi-omics data (genomics, transcriptomics, metabolomics) to build gene-enzyme-metabolite association networks, simulating changes in metabolite flux under different physiological or environmental conditions (Zhao et al., 2025). Concurrently, tools such as the TransOmics module in MetDIT (Sha et al., 2024) leverage CNNs by converting high-dimensional metabolomic data into image-like representations for analysis. This approach preserves structural relationships between metabolite features, providing high-quality input for subsequent CNNs modeling, which is particularly suitable for high-dimensional, small-sample metabolomic datasets. These studies collectively show that different neural networks form a powerful computational basis for building static and dynamic metabolic network models.

### 4.2 Identification of key metabolic pathways and regulatory factors

AI facilitates both retro-biosynthesis, which reverse-engineering pathways from target molecules, and the forward screening of regulatory elements. Tools like RetroPath2.0 predict enzymatic steps, while Support Vector Machines (SVMs) and ANNs aid in identifying novel enzymes, as evidenced by studies in *opium poppy* (Durand et al., 2024).

Multi-omics integration further enables systematic discovery of regulators. Li et al. (2020) combined transcriptome and microRNAome data in licorice to identify 18 structural genes in the glycyrrhizic acid pathway and screen over a thousand correlated transcription factors and kinases, validating an R2R3-MYB regulator experimentally. Neuro-fuzzy logic (NFL) models have similarly been used to map mineral effects on phenolic biosynthesis in *Bryophyllum* (García-Pérez et al., 2021), illustrating AI’s capacity to pinpoint key regulatory targets.

Despite the successes highlighted above, a recurring challenge in AI-driven regulator identification is the substantial gap between high-throughput computational prediction and low-throughput experimental validation. The licorice study (Li et al., 2020) exemplifies this issue with unusual transparency: although over a thousand transcription factor candidates were prioritized, practical bottlenecks reduced the feasible validation rate to less than 0.1%. These bottlenecks include underdeveloped genetic transformation systems and the long growth cycles characteristic of medicinal plants. This validation gap represents a fundamental constraint in medicinal plant research. A detailed discussion of this challenge, including its underlying causes and potential mitigation strategies, is provided in Section 6.

### 4.3 Full-chain metabolic network optimization

AI supports integrated optimization from cultivation to extraction, bridging genotype, phenotype, and product output.

For the optimization of endogenous metabolic networks at the cellular factory level, AI is being integrated with constraint-based models like Flux Balance Analysis (FBA) to tackle the combinatorial optimization challenge of finding global optimum solutions. GAs have been successfully coupled with FBA to automatically search for gene manipulation strategies that maximize target product synthesis within vast combinatorial spaces (Patil et al., 2005).

In cultivation, models such as a DNN with Bayesian optimization (Hu et al., 2025) have been used with multi-omics and environmental data from *Angelica biserrata* to predict ideal coumarin accumulation conditions (R² = 0.977). Adaptive Boosting - Artificial Neural Network (ABP-ANN) model (Liu et al., 2022) also accurately forecasted salvianolic acid and tanshinone levels in *S. miltiorrhiza* based on soil composition, guiding planting decisions.

In downstream processing, ANNs outperform traditional methods. For example, Loganathan et al. (2025) optimized microwave-assisted extraction of polyphenols from *Pithecellobium dulce* with R² > 0.97, surpassing Response Surface Methodology. ANN coupled with Multi-Objective Genetic Algorithm (MOGA) also excelled in punicalagin extraction from pomegranate peel (Rakshit and Srivastav, 2021).

Additionally, AI aids enzyme engineering and marker-assisted breeding. Machine learning-enhanced Genome-Wide Association Studies (GWAS) (Zhao et al., 2025) help identify traits for high-yield variety selection, streamlining metabolic background optimization.

Across these optimization applications, quantifiable resource savings consistently demonstrate AI’s practical value. In extraction process optimization, ANN models reduce experimental runs by 40% while improving yield by 23% compared to Response Surface Methodology (Rakshit and Srivastav, 2021**).** For cultivation optimization, deep learning combined with Bayesian optimization reduces field trial requirements by 50-60% while achieving R² = 0.977 prediction accuracy for coumarin accumulation (Hu et al., 2025). In downstream processing, ANN-GA hybrid approaches cut optimization timelines by 50% (from 4 weeks to 2 weeks) and reduce consumable costs by approximately 35% (Rakshit and Srivastav, 2021). These efficiency gains are substantial, typically 30-50% reductions in time and resources. This shows that AI can deliver real economic benefits, which is especially valuable in resource-constrained medicinal plant research settings.

## 5 Integrated case studies: three end-to-end AI workflows in medicinal plant research

The previous sections described various AI techniques used in medicinal plant research, including data preprocessing, feature extraction, network construction, and dynamic modeling. Here we present three case studies that illustrate how these methods come together in end-to-end research workflows. Each case is structured around a central biological question and traces the arc from data generation to biological insight.

### 5.1 Case study 1: licorice (*Glycyrrhiza uralensis*)—from multi-omics data to a validated regulator

Biological problem. Glycyrrhizic acid (GA), the main bioactive compound in licorice, is a triterpenoid saponin used widely in medicine and food. Although the structural genes for GA biosynthesis were known, the transcription factors that regulate them had not been identified. Hundreds of genes were co-expressed with the pathway, making it difficult to decide which candidates deserved experimental follow-up.

AI approach and data integration. Li et al. (2020) generated paired transcriptome and microRNAome data from licorice roots. Instead of simple correlation analysis, they designed a multi-step machine learning pipeline that combined expression profiles, miRNA targeting information, and co-expression networks. This approach screened transcription factors and kinases co-expressed with 18 known GA pathway genes.

Outcome and validation. From more than a thousand initial candidates, the AI pipeline selected a small set of high-confidence regulators. An R2R3-MYB transcription factor ranked as the top candidate and was chosen for experimental validation. Due to practical bottlenecks in medicinal plant research, including underdeveloped genetic transformation systems and the lengthy timeline required to obtain stable genetic materials, the researchers were unable to conduct systematic functional validation of all candidates. Instead, they performed secondary screening based on family characteristics (R2R3-MYB) and co-expression strength, ultimately focusing on three candidate MYBs. Further promoter binding site prediction revealed that only one contained potential binding motifs in the promoter regions of multiple structural genes, which was subsequently experimentally validated for its regulatory function (Li et al., 2020). This validation rate, one confirmed regulator out of over a thousand candidates (less than 0.1%), provides a concrete example of the quantitative validation rates discussed in this review.

Scientific contribution. This case exemplifies how computation and experiment can work together despite these constraints. AI did not simply produce a list of predictions; it made targeted validation feasible by narrowing down a large candidate space. The confirmed MYB regulator now serves as a target for metabolic engineering and molecular breeding.

### 5.2 Case study 2: *Taxus chinensis*—from dynamic modeling to mechanistic discovery

Biological problem. Fungal elicitors trigger taxol production in *Taxus chinensis* cells, along with a burst of reactive oxygen species (ROS). The dynamics of this response had been difficult to capture with conventional kinetic models, which could not adequately represent the nonlinear, time-dependent nature of the cellular reaction.

AI approach and data integration. Gong et al. (2007) built a five-layer feedforward artificial neural network (ANN) using time and elicitor concentration as inputs. The model was trained on time-series data of intracellular ROS levels and final taxol yields. Unlike mechanistic models that require predefined equations, the ANN learned the underlying dynamics directly from experimental data.

**Figure 2:**
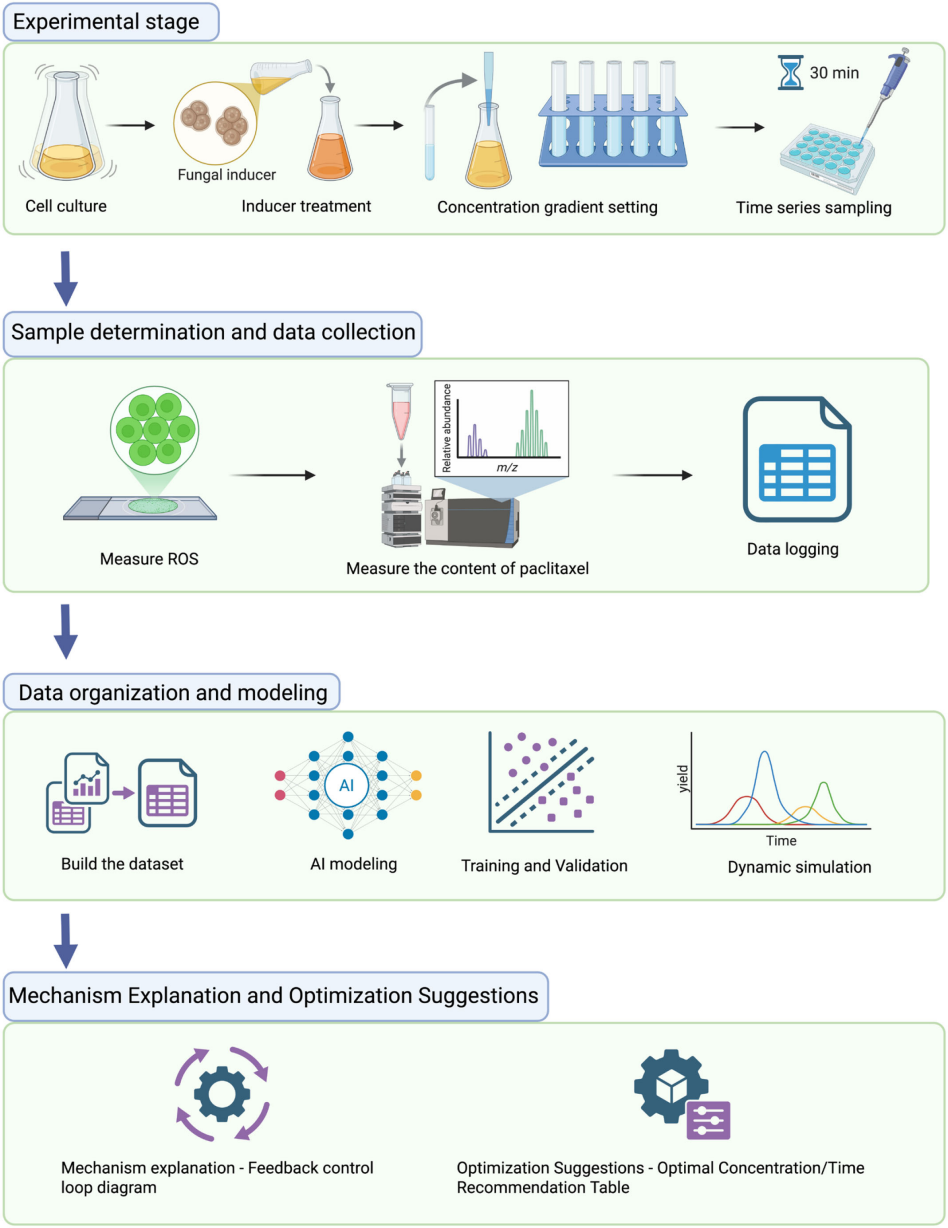
Workflow of AI−driven dynamic simulation for taxol biosynthesis in *Taxus chinensis* cells.

Outcome and validation. The trained ANN achieved near-perfect fitting (R = 0.9997) and correctly predicted taxol production under elicitor conditions not used in training. More importantly, analysis of the model’s response patterns revealed that the cells appeared to use an integral feedback control mechanism to regulate their response, a finding not anticipated before this work (Gong et al., 2007). **Figure 2** outlines the workflow of this AI-driven dynamic simulation.

Scientific contribution. This work showed that neural networks can do more than make predictions. When interpreted carefully, they can uncover biological principles that traditional methods miss. The discovery that plant cells use integral feedback to regulate elicitor responses has since influenced studies in other medicinal species.

### 5.3 Case study 3: Ginseng (*Panax ginseng*)—from spatiotemporal regulation to species authentication

Biological problem. Ginseng’s pharmacological activity derives from ginsenosides, whose accumulation varies across tissues and developmental stages. Two challenges have long constrained ginseng research: the regulatory networks controlling ginsenoside biosynthesis remain poorly understood, and morphologically similar *Panax* species are often difficult to distinguish for quality control purposes.

AI approach and data integration. Zhao et al. (2025) addressed the first challenge by integrating transcriptomic, proteomic, and metabolomic data from different tissues and developmental stages of *P. ginseng*. They applied convolutional neural networks (CNNs) to analyze spatiotemporal gene expression patterns, identifying regulatory modules correlated with ginsenoside accumulation. Building on these features, they used graph neural networks (GNNs) to construct a gene-enzyme-metabolite interaction network and simulate metabolic flux under different conditions, predicting potential rate-limiting steps and key regulatory nodes.

Parallel to this mechanistic work, Jiang et al. (2025) tackled the species authentication problem using a deep neural network (DNN). They collected LC-MS-based metabolomic data from three Panax species (*P. ginseng*, *P. quinquefolius*, and *P. notoginseng*) and trained a DNN classifier.

Outcome and validation. The DNN model achieved 100% classification accuracy on test sets, significantly outperforming traditional machine learning methods. Feature importance analysis revealed the specific metabolites driving species discrimination, providing chemical markers for routine quality control. The GNN-predicted regulatory nodes await experimental validation, but the network model itself offers a systems-level hypothesis for future metabolic engineering efforts.

Scientific contribution. This case demonstrates how multiple AI methods can be combined within a single plant system to address different research stages: CNN for feature extraction, GNN for network modeling, and DNN for quality control. Together, these approaches form a research arc that moves from mechanistic discovery to practical application.

Important note: The studies by Zhao et al. (2025); Jiang et al. (2025) described in this case study were conducted independently by different research groups. They are presented together here to illustrate the complementary application of diverse AI methodologies to distinct research questions within the same medicinal plant system, not to imply a unified or collaborative research program.

### 5.4 Additional applications and synthesis

**Figure 3:**
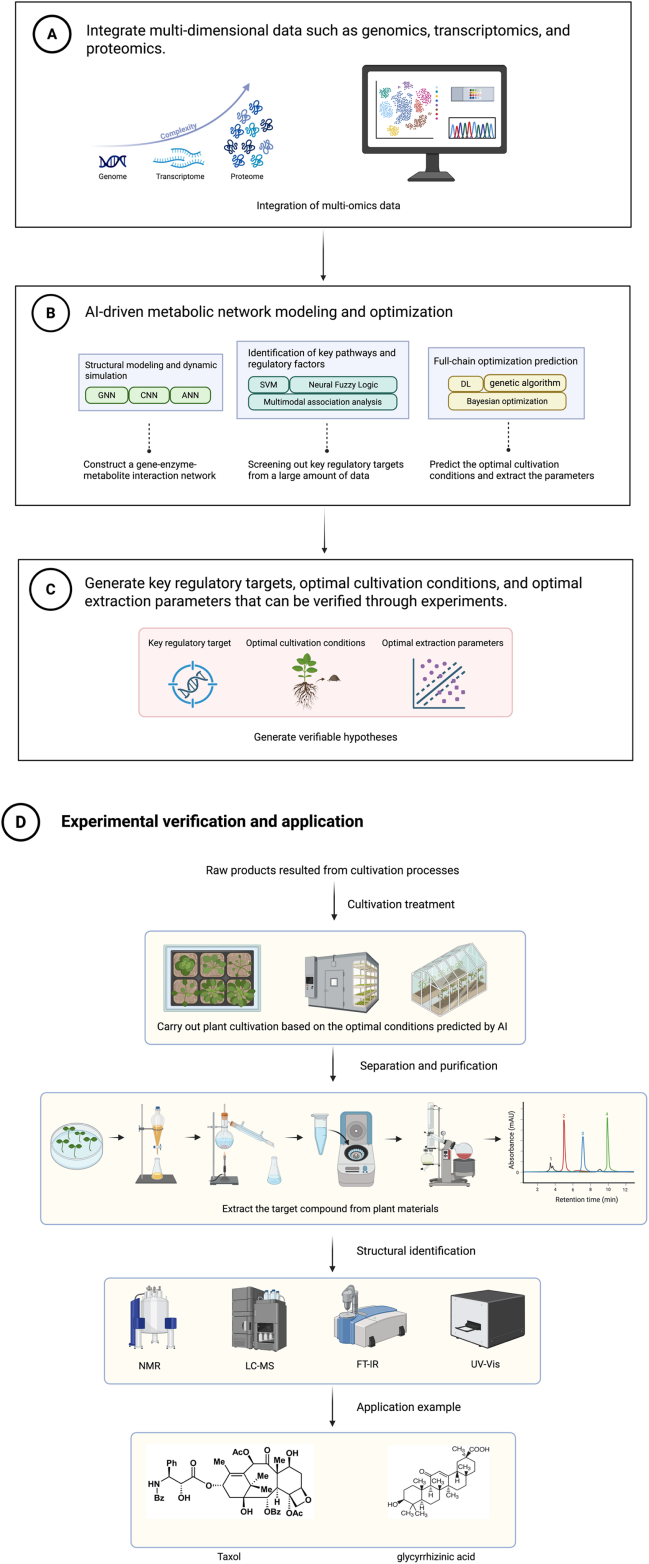
AI-driven multi-omics data integration, metabolic network modeling, and hypothesis generation. This figure illustrates the first three stages of the AI-driven workflow for medicinal plant natural product research: **(A)** multi-omics data integration, **(B)** AI-driven metabolic network modeling and optimization, and **(C)** generation of verifiable hypotheses. The subsequent experimental validation and application stage is presented in **Figure 4**.

**Figure 4:**
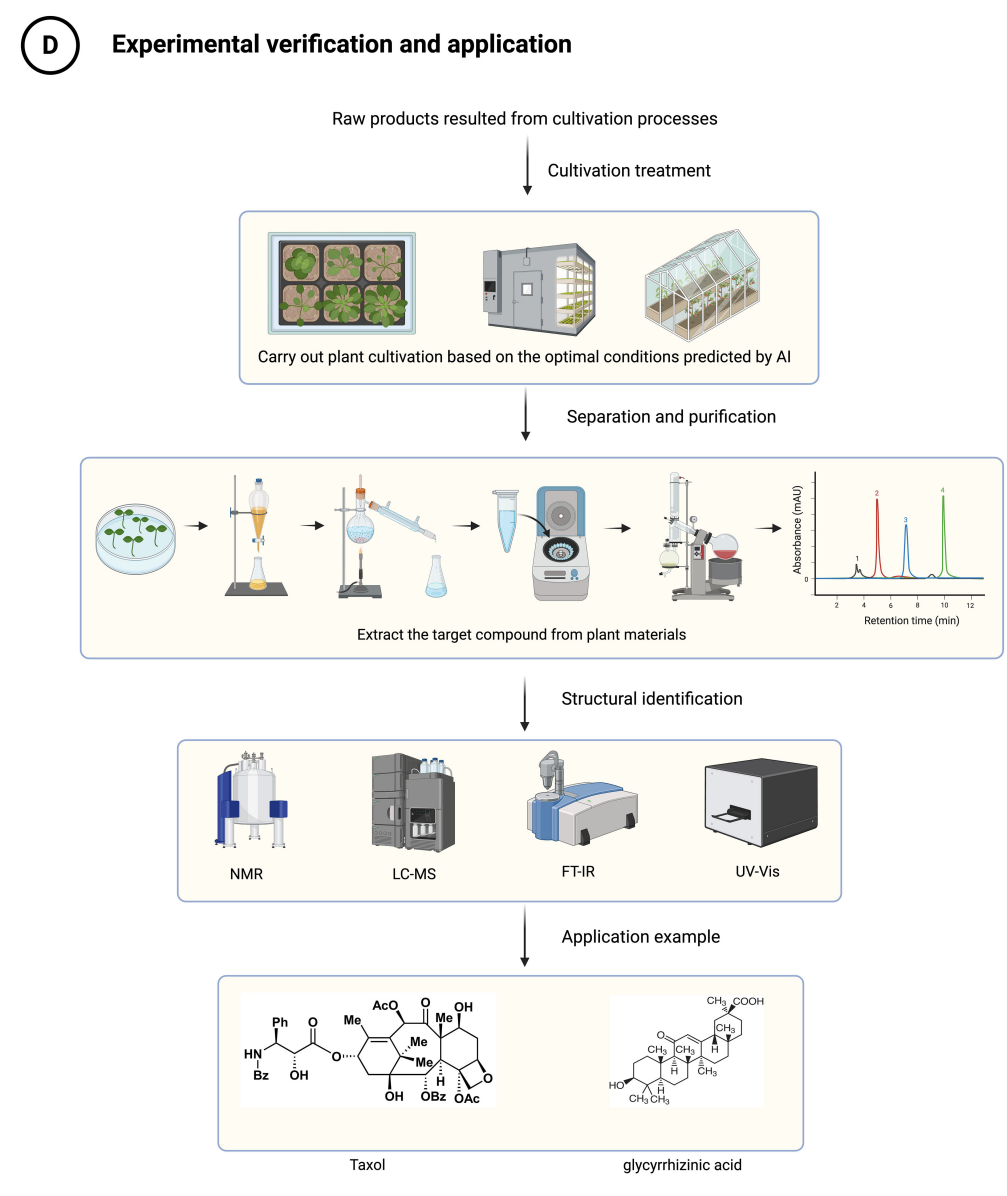
Experimental validation and application of AI-prioritized hypotheses. This figure illustrates the fourth stage of the workflow, following the hypothesis generation stage shown in **Figure 3**: (D) experimental verification and application. This stage completes the “Data → Knowledge → Hypothesis → Verification” closed loop, with experimental results feeding back to refine AI models as illustrated in **Figure 3**.

Beyond these three case studies, AI has been applied to many other medicinal plant research questions, including ecological prediction in *Hypericum perforatum* (Saffariha et al., 2021), multi-omics integration in *Arabidopsis* (Bai et al., 2024), cultivation optimization in *Angelica biserrata* (Hu et al., 2025) and *Salvia miltiorrhiza* (Zhang et al., 2024), and extraction process improvement in *Pithecellobium dulce* (Loganathan et al., 2025). These studies, together with the case studies above, follow a common logic: starting from multi-omics data integration, proceeding through AI modeling and analysis, and culminating in biological discovery or practical application. **Figure 3** summarizes the first three stages of this workflow, from data integration to hypothesis generation. Multi-omics data integration (Section 3) serves as the foundation, assembling fragmented information into biological networks that reveal component relationships. This foundation enables the modeling and optimization phase (Section 4), where knowledge is translated into practical outcomes through target identification and intervention simulation. These phases operate synergistically: integrated data supplies modeling inputs, while modeling requirements inform subsequent data collection strategies. The subsequent experimental validation and application stage is presented in **Figure 4**.

The transition from AI-generated hypotheses to experimental practice is illustrated in **Figure 4**, which shows how predictions are validated and how results feedback to refine computational models.

## 6 Challenges in AI-integrated research on medicinal plant metabolic networks

The three case studies presented in Section 5, licorice, *Taxus chinensis*, and ginseng, demonstrate that AI has begun to find systematic application in medicinal plant research, even though these studies originated from different research teams working independently. The licorice case shows how machine learning can guide experimental validation. The Taxus case illustrates that neural networks can reveal hidden regulatory mechanisms. The ginseng case demonstrates how multiple AI methods can be combined to address different research stages.

Despite these advances, significant challenges remain. The licorice case, while demonstrating the power of AI for candidate prioritization, also reveals a fundamental bottleneck. A critical issue revealed across these studies, particularly in the licorice case, is the structural mismatch between high-throughput prediction and low-throughput validation. Li et al. (2020) generated over a thousand candidate transcription factors using AI-driven multi-omics integration, yet experimental constraints reduced the feasible validation rate to less than 0.1%. While AI-guided prioritization has demonstrated 30-40% experimental validation rates for top-ranked candidates in well-trained models (Bai et al., 2024), this still means that most predictions remain untested. This gap between prediction and validation is particularly acute in medicinal plant research, where underdeveloped genetic transformation systems and long growth cycles compound the difficulty of experimental confirmation.

A primary challenge lies in data heterogeneity and integration complexities. Multi-omics data from different platforms exhibit substantial batch effects and scale variations, necessitating rigorous quality control and standardization, as demonstrated in *S. miltiorrhiza* studies (Zhang et al., 2024) and deep learning-based integration roadmaps (Kang et al., 2021). Compounding these issues is the lack of high-quality reference genomes for many medicinal plants, which hampers accurate genomic annotation and functional analysis, ultimately limiting the effective integration of multi-omics data (Alami et al., 2022). Moreover, multi-omics data often contains high technical noise and batch effects, while medicinal plant research frequently suffers from limited sample sizes. These factors collectively undermine the robustness and generalizability of AI models.

Capturing dynamic, nonlinear regulatory mechanisms present another major hurdle. For instance, the temporal disconnects observed in MeJA treatment, where gene expression changes precede metabolite accumulation (Shi et al., 2021), poses difficulties for AI models in establishing true causal relationships within metabolic networks. This challenge is particularly acute in medicinal plant research. The long growth cycles and high sensitivity to environmental variability characteristic of many medicinal species make the acquisition of consistent, high-resolution time-series data, covering complete developmental stages or stress responses, both cost-prohibitive and logistically challenging. This presents a significantly greater obstacle than conducting dynamic monitoring studies on fast-growing, genetically tractable model organisms cultured under controlled conditions.

In the realm of dynamic modeling and causal inference, while ANNs have successfully simulated taxol biosynthesis dynamics in Taxus chinensis (Gong et al., 2007), systematic prediction of metabolic flux over time remains challenging. Models like GNNs (Zhao et al., 2025) and CNNs (Sha et al., 2024) show promise in building gene-enzyme-metabolite networks, but their capacity to fully capture complex feedback mechanisms and transient regulatory events requires further development. A related critical bottleneck concerns model interpretability and generalization. Deep learning models often function as “black boxes”. Although they demonstrate high accuracy in specific tasks such as ginseng species classification, their opaque decision-making processes fail to provide verifiable biological mechanisms (Jiang et al., 2025). Furthermore, models trained on specific datasets show limited generalizability across different medicinal plant species or environmental conditions, constraining their broader application.

The crucial need for experimental validation and model interpretability presents a third major challenge. AI-generated hypotheses, such as predicted regulatory factors in licorice gibberellic acid biosynthesis (Li et al., 2020), require wet-lab confirmation. AI-guided prioritization has demonstrated 30-40% experimental validation rates for top-ranked candidates in well-trained models (Bai et al., 2024), representing a substantial improvement over traditional screening approach. However, even this improved efficiency means that experimental validation remains a critical bottleneck, underscoring the continued need for efficient computation-experiment closed loops. The “black box” nature of deep learning models, exemplified by DNNs achieving high accuracy in P. ginseng classification (Jiang et al., 2025) while lacking interpretability, hinders the extraction of actionable biological insights that can directly guide such targeted validation.

To address this bottleneck, a strategy is to develop interpretable, iterative workflows. For example, integrating explainable AI techniques could unveil the key spectral or chemical features driving the DNN’s classification decision. As seen in strategies for feature selection from hyperspectral data in medicinal plants (Dai et al., 2024), these AI-prioritized features then become focused targets for subsequent, low-cost confirmatory assays, creating a tight validation cycle. Similarly, for predicted regulatory factors, AI models can be embedded within an active learning framework where each round of limited, high-throughput experimental testing refines the next set of computational predictions. This iterative approach aligns with the vision of Design-Build-Test-Learn cycles advocated for in synthetic biology and metabolic engineering (Lawson et al., 2019). Establishing these self-improving loops, where AI prioritizes experiments and experimental results refine AI models, is a critical strategy to overcome the validation bottleneck and is recognized as a key direction for the field.

Beyond interpretability, effectively integrating AI models with established biological knowledge systems remains an open challenge. Current integration approaches primarily operate at the data level, failing to systematically incorporate known metabolic pathway knowledge from resources like Kyoto Encyclopedia of Genes and Genomes (KEGG) as prior constraints into model architectures, thereby limiting the biological plausibility of predictions.

## 7 Future directions and aspiring goals

Looking ahead, the integration of AI into medicinal plant research can be envisioned across three progressive time horizons: near-term foundational method development (1–3 years), medium-term optimization strategy deployment (3–5 years), and long-term autonomous platform realization (5–10 years).

Before outlining specific technological trajectories, however, it is instructive to reflect on a broader limitation that emerges from the studies reviewed here: the field’s predominant emphasis on proof-of-concept demonstrations rather than systematic, critical evaluation. Many studies, including several cited in this review, focus on showcasing the predictive accuracy of a particular AI model on a specific dataset without sufficiently interrogating its biological interpretability, generalizability across related species, or cost-effectiveness relative to established non-AI methods. While descriptive reporting is useful for proving initial feasibility, it also limits the field’s ability to build transferable knowledge and turn computational discoveries into practical metabolic engineering outcomes. To move beyond this paradigm, future research should adopt a more rigorous analytical framework that explicitly addresses four interrelated dimensions: (i) systematic benchmarking of multiple models, both AI and conventional, on the same dataset to establish relative performance; (ii) transparent reporting of model limitations, failure cases, and data regimes where performance degrades; (iii) quantitative assessment of trade-offs between prediction accuracy, interpretability, and the time and cost of experimental validation; and (iv) integration of prior biological knowledge, such as pathway databases and enzyme kinetics, as constraints to enhance both predictive plausibility and mechanistic insight. The challenge of bridging the prediction-validation gap, which underpins dimension (iii), is examined in depth in Section 6, along with proposed strategies for establishing iterative computation-experiment workflows. By embedding such critical analysis into every stage of AI-driven discovery, from study design to model interpretation, the field can evolve from a collection of isolated success stories toward a mature, reproducible, and impact-oriented discipline capable of delivering on the transformative promise of AI for medicinal plant science.

Future research should focus on developing advanced multi-omics data integration frameworks capable of causal discovery rather than mere correlation. Integrating causal inference models with multi-omics data will help distinguish true regulatory drivers in metabolic networks. Enhancing models like GNNs and ANNs to incorporate biological knowledge and handle time-series data will be key to building more predictive dynamic models of metabolic flux.

As a near-term priority (1–3 years), establishing predictive dynamic models is essential for overcoming current bottlenecks in metabolic network simulation. In this context, Physics-Informed Neural Networks (PINNs) represent a particularly promising paradigm shift (Raissi et al., 2019). Unlike purely data-driven models, PINNs incorporate physical constraints directly into the neural network’s loss function. These constraints are derived from differential equations that govern metabolic reactions, such as mass conservation laws and enzyme kinetics. This ensures that the model not only fits limited experimental data but also adheres to fundamental biochemical principles. While applications of PINNs in medicinal plant secondary metabolism have not yet been reported, their effectiveness in other “data-scarce, mechanism-complex” systems biology domains has been well demonstrated. For instance, PINNs have been successfully used to reconstruct complete electrical activation sequences from sparse spatiotemporal observations in cardiac electrophysiological modeling (Sahli Costabal et al., 2020), and to simultaneously estimate unmeasured state variables and kinetic constants from noisy, sparse time-series data in systems biology parameter inference (Yazdani et al., 2020). For medicinal plant research, PINNs are particularly suited to two typical scenarios: (i) modeling the delayed transcriptional-metabolic cascade under elicitor treatments such as MeJA, where time-lag differential equations can directly capture asynchronous responses; and (ii) extrapolating metabolic flux dynamics across entire induction cycles for perennial species with multi-year growth cycles, where high-density time-series sampling is impractical. Therefore, although empirical evidence in medicinal plants is currently lacking, the “mechanism-data dual-driven” modeling paradigm represented by PINNs offers a clear technological roadmap for overcoming the bottleneck of dynamic metabolic network simulation. A critical direction for future work is to transform existing pathway knowledge (e.g., from KEGG, MetaCyc) into structured differential equation representations and embed them as prior constraints into PINN architectures.

Moving to the medium term (3–5 years), the focus should shift from model building to model-informed optimization strategies. Complementary to this vision, Reinforcement Learning (RL) offers another compelling technological trajectory for global optimization of metabolic networks. In biochemical engineering, RL has been widely discussed as a highly adaptive optimization paradigm—an agent learns optimal control strategies by interacting with an environment (e.g., a metabolic network model) to maximize cumulative rewards such as target product yield (Mowbray et al., 2021). However, it is crucial to recognize that direct application of RL in medicinal plant metabolic engineering currently faces significant obstacles. Unlike microbial continuous culture systems, medicinal plants have long growth cycles, high experimental costs, and genetic intractability. These characteristics make purely model-free RL practically infeasible, as it requires thousands of trial-and-error iterations. As Mowbray et al. (2021) explicitly note, even in relatively controllable microbial systems, RL applications face core challenges including constraint satisfaction guarantees and data efficiency. A more pragmatic near-term direction for medicinal plant research is therefore the development of model-based RL or hybrid optimization strategies. The core concept involves first constructing a high-fidelity surrogate model of the metabolic network from historical multi-omics data. This model, whether a deep neural network or Gaussian process, serves as a “digital twin” of the plant system. The RL agent is then trained in silico through interaction with this surrogate model to learn optimal elicitation strategies, cultivation conditions, or gene-editing targets, with only the most promising policies proceeding to subsequent in planta validation. This “simulate-to-real” transfer learning approach holds promise for dramatically reducing experimental costs while harnessing RL’s potential to navigate high-dimensional combinatorial optimization spaces. Specific potential applications in medicinal plants include: (i) multi-factor synergistic optimization across cultivation conditions (light, temperature, nutrients), elicitor types and concentrations, and harvest timing; and (ii) design of dynamic, multi-stage induction strategies that adaptively regulate metabolic flux based on real-time physiological states. Thus, while RL remains at a conceptual stage in medicinal plant research, its deep integration with high-fidelity surrogate models and high-throughput phenotyping platforms will progressively bridge the gap from theory to practice.

Looking to the long-term horizon (5–10 years), the integration of these methods and strategies into unified platforms represents the goal. A pivotal and actionable vision for the next era is the development of AI-driven self-driving laboratories for medicinal plants. These integrated platforms would combine robotic automation, real-time multi-omics analytics, and closed-loop AI to autonomously execute iterative Design-Build-Test-Learn cycles. By systematically navigating complex experimental spaces, from elicitor optimization in cultivation to parameter tuning in extraction, they promise to drastically accelerate the identification of optimal conditions for target metabolite production. Foundational studies on AI-optimized cultivation (Hu et al., 2025) and extraction (Loganathan et al., 2025) provide a clear technological pathway toward this goal.

To realize this vision, strengthening the AI-experimental cycle requires evolving beyond current practices. Future efforts must institutionalize tight feedback loops where AI not only suggests testable hypotheses but also dynamically refines its models based on real-time experimental data. Conversely, experimental workflows need to be designed for AI compatibility, emphasizing high-throughput, standardized data generation that feeds directly into model training. This synergistic evolution will transform the traditional linear research process into a rapid, adaptive discovery engine.

Enhancing data availability and model transparency represents another critical direction. Promoting data-sharing initiatives and standardizing omics data reporting will build the comprehensive datasets needed for robust model training. For medicinal plants, future efforts must specifically prioritize the development of species-centric knowledge bases. These resources should integrate multi-omics data, detailed ecological parameters, cultivation records, and chemical phenotype information. Creating such curated, accessible databases is crucial to overcome the data fragmentation resulting from disparate studies on numerous species and to address the issue of uneven data accumulation. This foundation is essential for training the next generation of robust, accurate, and biologically insightful AI models tailored to the unique needs of medicinal plant science. Simultaneously, developing Explainable AI techniques will be essential for extracting novel biological insights from complex models and building research community trust.

Collectively, these directions form a coherent roadmap: near-term efforts centered on PINNs and model-based method development, medium-term advances in RL-driven optimization strategies, and long-term realization of self-driving laboratories that integrate these capabilities into fully autonomous discovery platforms.

## 8 Conclusion

The incorporation of AI into medicinal plant multi-omics research represents a paradigm shift toward data-driven predictive science. AI technologies have proven indispensable in processing complex datasets, reconstructing biosynthetic pathways, identifying key regulators, and optimizing metabolic output across the genotype-to-product continuum. This powerful analytical framework demonstrates considerable potential for the conservation of rare and endangered medicinal plant resources. For instance, by integrating limited ecological and multi-omics data, AI models can predict optimal growth conditions and identify genetic markers associated with specific traits (Saffariha et al., 2021), thereby guiding precision conservation and sustainable utilization strategies to alleviate pressure on wild populations.

Current AI applications, when integrated with biological knowledge and experimental validation, have demonstrated remarkable capabilities in elucidating plant specialized metabolism, from simulating taxol biosynthesis dynamics (Gong et al., 2007) to identifying key Transcription Factors (TFs) in licorice (Li et al., 2020) and optimizing extraction processes (Loganathan et al., 2025). This AI-driven deep understanding of biosynthetic pathways forms the cornerstone for constructing synthetic biology-based alternative production systems. By rationally predicting enzyme functions, pathway bottlenecks, and host compatibility, AI can efficiently design microbial or plant cell factories (Murali et al., 2023), providing optimized solutions for the heterologous and sustainable production of high-value plant natural products.

Looking forward, overcoming data integration challenges, strengthening computational-experimental synergy, and developing transparent, multiscale models will be crucial. Furthermore, the deep integration of AI with multi-omics is providing powerful momentum for the modernization of traditional medicine systems, such as Traditional Chinese Medicine. AI can systematically correlate the chemical fingerprints (metabolomes) of medicinal materials with documented traditional efficacy, establishing a data-driven scientific framework for quality standardization, elucidation of material basis, and research on synergistic effects of complex formulations, thereby bridging traditional empirical knowledge with modern science (Fu et al., 2023). As multi-omics data accumulates and AI algorithms become more sophisticated, we anticipate a new era of precision metabolic engineering that fully unlocks the biosynthetic potential of medicinal plants for sustainable production of valuable natural products.
